# Early intervention for preventing posttraumatic stress disorder: an Internet-based virtual reality treatment

**DOI:** 10.3402/ejpt.v6.25608

**Published:** 2015-04-02

**Authors:** Sara A. Freedman, Ehud Dayan, Yael Bleich Kimelman, Heidi Weissman, Renana Eitan

**Affiliations:** 1 School of Social Work, Bar Ilan University, Ramat Gan, Israel; 2 Department of Psychiatry, Hadassah University Hospital, Jerusalem, Israel; 3 Sonarion Ltd., Jerusalem, Israel

**Keywords:** Internet-based therapy, cognitive behavior therapy, PTSD, early intervention

## Abstract

**Background:**

Posttraumatic stress disorder (PTSD) develops in approximately 20% of people exposed to a traumatic event, and studies have shown that cognitive-behavioral therapy (CBT) is effective as a treatment for chronic PTSD. It has also been shown to prevent PTSD when delivered early after a traumatic event. However, studies have shown that uptake of early treatment is generally low, and therefore, the need to provide interventions through other mediums has been identified. The use of technology may overcome barriers to treatment.

**Objective:**

This paper describes a randomized controlled trial that will examine an early CBT intervention for PTSD. The treatment incorporates virtual reality (VR) as a method for delivering exposure-based elements of the treatment. The intervention is Internet based, such that the therapist and patient will “meet” in a secure online site. This site will also include multi-media components of the treatment (such as videos, audios, VR) that can be accessed by the patient between sessions.

**Method:**

Two hundred patients arriving to a Level 1 emergency department following a motor vehicle accident will be randomly assigned to either treatment or control groups. Inclusion criteria are age 18–65, PTSD symptoms 2 weeks posttrauma related to current trauma, no suicidality, no psychosis. Patients will be assessed by telephone by a team blind to the study group, on four occasions: before and after treatment, and 6 and 12 months posttreatment. The primary outcome is PTSD symptoms at follow up. Secondary outcomes include depression and cost effectiveness. Analyses will be on an intention-to-treat basis.

**Discussion:**

The results will provide more insight into the effects of preventive interventions, in general, and Internet-based early interventions, in particular, on PTSD, in an injured population, during the acute phase after trauma. We will discuss possible strengths and limitations.

Posttraumatic stress disorder (PTSD) (American Psychiatric Association [APA], 2013) was once considered a refractory disorder but advances over the past two decades now provide patients with a variety of effective treatment options. Much research has focused on trauma-focused cognitive-behavioral therapy (CBT), which is more effective than other psychotherapies in reducing chronic PTSD, that is, PTSD that has lasted for more than 3 months (APA, 2013; Bisson et al., 2007).

Studies have shown that while most people exhibit symptoms shortly following trauma exposure, the vast majority will recover naturally (Freedman, Brandes, Peri, & Shalev, 1999). This has led to the understanding that the optimal time for intervention may be shortly after trauma exposure (Kearns, Ressler, Zatzick, & Rothbaum, 2012). A growing body of research indicates that it is possible to provide effective therapeutic interventions in this post-exposure time-period for symptomatic patients. These studies report that CBT is more effective than other treatment modalities (Bryant, Sackville, Dang, Moulds, & Guthrie, 1999), waitlist control (Sijbrandij et al., 2007), and medication (Shalev et al., 2012) in preventing PTSD. Main studies have shown that the effects found can be long lasting, with some follow-up studies showing that some patients that receive CBT remain symptom free for up to 4 years (Bryant, Moulds, & Nixon, 2003) although this is not universal.

These studies have also highlighted, however, that most people avoid treatment (Shalev, Ankri, Peleg, Israeli-Shalev, & Freedman, 2011). These individuals may not come for treatment for a variety of reasons, including stigma, concern over time lost from work, lack of locally available therapists, and lack of sufficient resources in post-disaster settings (Hoge et al., 2004).

Novel treatment modalities for PTSD treatment which can help overcome these barriers are still needed, and such platforms may help attract people to treatment who may not otherwise participate (Kazdin & Blase, 2011). The Internet as a delivery method for interventions may help in overcoming barriers to treatment and assessment, since it does not require travel time to and fro a therapy session, does not involve the stigma of presenting oneself to a department of psychiatry, and is able to overcome the issue of locally available therapists. There is a growing evidence-base concerning the successful application of Internet-based therapy for treating chronic PTSD (Klein et al., 2010; Knaevelsrud & Maercker, 2010; Lange et al., 2003; Ruwaard, Broeksteeg, Schrieken, Emmelkamp, & Lange, 2010). One study has examined an Internet-based early intervention for PTSD; results indicate that take up of treatment was relatively low, and no significant differences were found between those who received the treatment compared to those who did not (Mouthaan et al., 2013).

Internet-based treatments take many forms; two common ones have been discussed here. The first is a traditional website, where patients log in and work through a number of tasks as directed on the website. Sometimes these are backed up with email contact with the therapist. For instance, in Interapy (Lange et al., 2003) patients are asked to write essays, which are then read and commented on by the therapist.

The second format is “cybertherapy,” where the therapist and patient “meet” in cyberspace, for example, using the Second Life 3-D virtual world (www.secondlife.com). This world allows participants to utilize avatars that can “live” in the virtual world, meeting people and carrying out a range of activities. There are a number of therapy options in the virtual world, and the therapist and patient mimic a normal face-to-face meeting in so far as they are both present in real time. However, the therapist need not be in the same country or even time-zone as the patient, and the therapist is also able to utilize online recourses such as readings or videos. The Second Life platform is already being used as a tool for education about health and other health-related activities, as well as for anxiety (Lisetti et al., 2009).

A different direction regarding novel modalities concerns the use of virtual reality (VR), which has been increasingly utilized in the treatment of anxiety disorders (Meyerbröker & Emmelkamp, 2010). A significant aspect of CBT treatments for anxiety disorders includes exposure to feared situations. VR allows the therapist and patient total control over the feared stimuli, thus making the exposure easier to administer. In addition, VR worlds that recreate the scene of the event enable the process of re-telling the trauma narrative. A pilot study conducted by this team has shown that PTSD can be successfully treated with CBT augmented by exposure within a VR environment (Freedman et al., 2010). Other studies have also shown that chronic PTSD from a variety of traumatic events (e.g., war in Afghanistan, World Trade Center) can be successfully treated with VR (Difede et al., 2007; Difede & Hoffman, 2002; Rothbaum, Hodges, Ready, Graap, & Alarcon, 2001). Until now, VR treatment has taken place in the traditional setting of the therapist's office.

## Current study

**Fig. 1:**
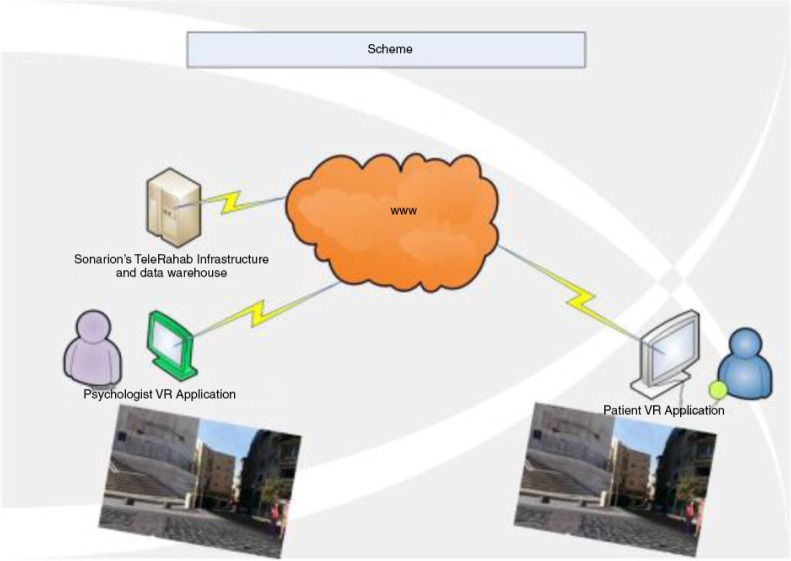
Virtual Reality environment.

The intervention used in this study—i-VR (Internet-VR)—was specifically designed as an intervention for PTSD that combines the two platforms described here, creating a VR environment that can be accessed via the Internet. Both the therapist and the patient will concurrently occupy the environment, even though they are not in the same physical place. This is illustrated in Fig. 1. The i-VR platform will be easier to access than other cyberspace environments. The intervention includes evidence-based components for PTSD, such as breathing retraining, exposure, and cognitive restructuring. It is a five session protocol, similar to those that have been used in other early intervention studies for PTSD (Bryant et al., 1999). This randomized controlled trial (RCT) will examine the following hypotheses:

1. i-VR CBT will be more effective at reducing PTSD symptoms than the waitlist control, such that they will have lower PTSD levels at all follow-up assessments.
2. i-VI CBT will result in less use of health services and fewer missed work days than the waitlist control.
3. Patients who spend more time using the i-VR environment between sessions will benefit more from the treatment.

## Method

### Participants

Adult patients, aged 18–65, who arrive at a Level I emergency department (ED) following a motor vehicle accident (MVA) will be recruited to the study. Inclusion criteria were the ability to give informed consent and PTSD symptoms 2 weeks posttrauma; this will be assessed regarding all symptoms apart from the criteria of time. Exclusion criteria include suffering from, or have suffered from in the past, bipolar disorder, psychotic disorder, or substance abuse (as measured by the SCID); suffering from ongoing PTSD (as measured by the CAPS); loss of consciousness on arrival to the ED; suffering from another Axis I anxiety disorder that requires immediate treatment; and currently presents as a suicide risk.

### Study design

In this RCT, patients will be randomized to one of two conditions. The first is the i-VR treatment, consisting of five sessions; the second is waitlist control. The study has received IRB from Hadassah Hospital (HMO 0056-013); its ClinicalTrials.gov identifier is NCT01760213. Patients will sign informed consent, and be able to leave the study at any point. Patients still symptomatic at the end of the research will be referred to the Outpatient Psychiatry Department, Hadassah Hospital. The Internet environment ensures patient confidentiality. All data will be stored with subject numbers only, without revealing identify of the patients.

### Intervention

The treatment protocol was created by SAF and ED, based on their previous work in this field. It consists of five sessions. All interventions described will be available in multi-formats: the therapist's verbal explanation, a written text that will appear in the “therapy room,” animation of examples and metaphors used, video clips, and VR scenarios. All these will be available between sessions for the patient to use. For example, breathing retraining will be explained verbally by the therapist, there will be a text of this explanation available, as well as a video. In the initial session, the therapist and the patient meet in a virtual therapy room. The therapist builds rapport with the patient, provides education regarding both common reactions following traumatic events and the therapy, and agrees a problem list. At the end of the session, the therapist teaches the patient anxiety-reducing breathing retraining. In the second session, the rationale for in-vivo exposure is given—the patient learns why fear reduces when exposed to avoided, although safe, stimuli. The therapist and the patient then build a hierarchy of avoided situations. Once a situation is chosen, the patient will carry out exposure using the library of images and media components that are stored in the i-VR environment. The therapist and the patient will monitor subjective units of distress ratings. The patient will continue this exposure task in the i-VR space as homework. In the third and fourth sessions, the rationale for building a trauma narrative is given, and the patient will narrate the trauma using the VR components of the environment. This will include five levels of difficulty: a local street with light traffic; a local street with two cars that nearly crash; fast road, with overtaking; accident without sounds/difficult sights; accident with crash sounds, sirens, screaming, glass, and blood. In the fourth session, the patient is introduced to the importance of thoughts and the therapist shows that patient how to record his or her thoughts. In the final session, the patient and the therapist work on thought challenging, discuss the intervention, identify areas that the patient should continue to work on, and discuss techniques for relapse prevention.

The i-VR platform enables both the patient and the therapist to interact in real time. It is private and secure, and easy to access from a personal computer, requiring no complex navigation. The VR environment will be constructed individually for each patient, using images that relate to their personal traumatic event. As a result, each patient will be able to meet his or her therapist and recreate the traumatic event in an environment that is customized.

The therapist has control of functionality so that he or she can control the patient experience and the treatment flow. This control can be delegated to the patient, thus allowing the therapist to see where the patient chooses to go in the i-VR space, and what he or she chooses to see. The session will be recorded, thus enabling replay and post-analysis of the user movement in i-VR space. The therapist will be able to utilize pop-up images and videos at a chosen location in the i-VR scene, and also to play 3D sounds at chosen spots within the scene, enabling the patient to hear the sound in relation to the distance he or she chooses to be from that sound, so that the nearer the patient gets the stronger the sound will be.

**Fig. 2:**
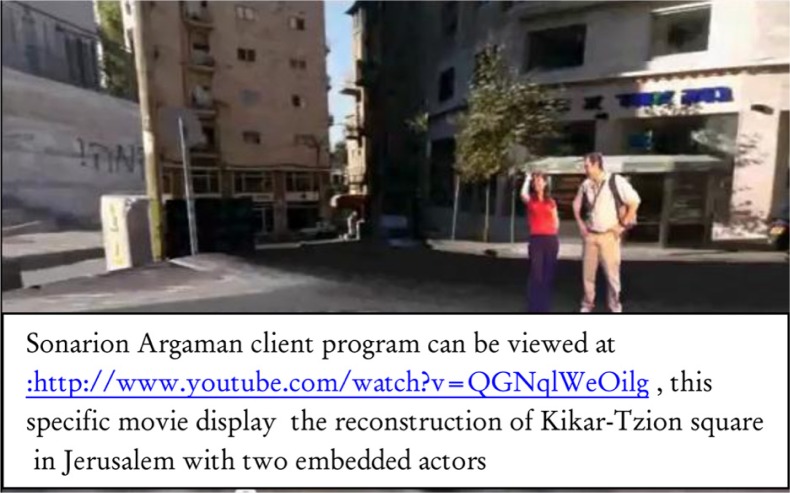
Example of an i-VR scene.

The patient will be able to navigate independently in the i-VR scene or view the scene as the therapist guides him or her through. The patient will be able to practice independently and view a replay of his or her previous session. The practice data will be stored in a database so the therapist will be able to monitor the patient's offline independent self-work. An example of an i-VR scene is shown in Fig. 2.

### Procedure

The telephone interview team will call suitable ED patients within 2 weeks of their traumatic event, and an initial telephone assessment will determine whether the individual indeed meets study criteria. After receiving verbal informed consent, subjects will be given the details of a secure Internet site, where they will be asked to fill out questionnaires. Subjects with a PCL-5 score over 33 will be telephoned for a second telephone assessment. During this assessment, the patient will be assessed with the Structured Clinical Interview for DSM-IV (SCID I) and Clinician-Administered PTSD Scale (CAPS-5). If the patient fulfills the inclusion criteria, the patient will be invited to join the RCT. Subjects who enter the trial will receive a detailed description of the study, and if interested in participating, they will sign informed consent. These clinical interviews will be carried out by the telephone interview team, who will remain blind to treatment condition.

Patients will be randomly assigned by computer to one of the two treatment arms. Assigning the participants to either treatment or control group will be done using block randomization to ensure comparable numbers of participants of same gender and same initial PTSD level in each group. Patients in the waitlist condition will fill out questionnaires via the secure Internet site once a week. These will be checked by a team member dedicated to this task, who will be able to contact these patients should a specific need arrive. Patients in the i-VR arm will receive a laptop computer, with the i-VR program loaded on. They will receive training on how to access it, and a time for the first i-VR session will be made. The five i-VR sessions will be with experienced CBT therapists; these are all clinical psychologists or clinical social workers, who have received postgraduate certification as CBT therapists. In addition, they are trained in trauma-focused CBT and have extensive experience in protocol-driven research trials. Therapists will receive weekly supervision from the PI.

Sessions will be taped with the consent of the patient, and this will be used for supervision purposes. In addition, a random 10% of sessions will be evaluated by external supervisors for fidelity to the treatment protocol.

**Fig. 3:**
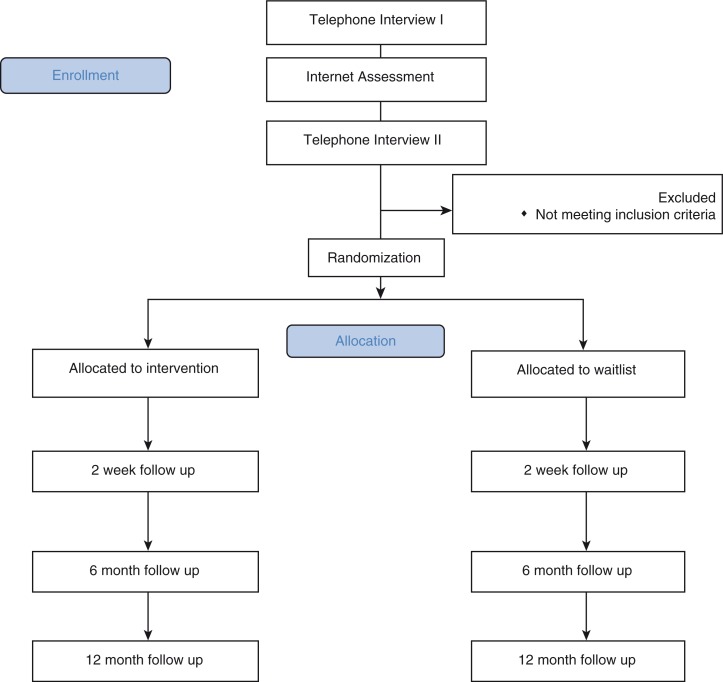
CONSORT diagram.

Follow-up assessment will be conducted by the telephone interview team through clinical telephone interviews, at 2 weeks following the end of treatment, and again 6 and 12 months later (Fig. 3).

### Research tools

*Self-Report Questionnaires*: These will be administered via a secure Internet site.

1. BDI II: The Beck Depression Inventory II (Beck, Steer, & Brown, 1996) is a 21-item self-report questionnaire that has been widely used as a measure of depressive symptoms in trauma populations.
2. PCL-5 (Weathers, Litz, et al., 2013): This is a 20-item self-report questionnaire, assessing PTSD symptoms according to DSM 5 (APA, 2013). A cut off score of 33 (Weathers, Litz, et al., 2013) indicates probable PTSD.

*Clinical interviews*: Patients will be assessed by telephone, before treatment and after treatment, and 6 and 12 months following the end of treatment. These interviews will be carried out by trained psychologists, who will remain blind to treatment condition. The following instruments will be used:

1. SCID I: It (First, Spitzer, Gibbon, & Williams, 1996) is used to evaluate the current and lifetime presence or absence of Axis I disorders. The Hebrew version has been used in many studies. The SCID is considered to be the gold standard when evaluating Axis I disorders.
2. CAPS-5: It (Weathers, Blake, et al., 2013) is a 30-item semistructured interview that confers a diagnosis of PTSD according to DSM 5 (APA, 2013) and a continuous measure of PTSD symptoms’ frequency and intensity.
3. BDI: The Beck Depression Inventory (Beck et al., 1996) as described above. This will be administered as an interview.
4. The Trimbos/iMTA questionnaire for Costs associated with Psychiatric illness, TiC-P (Van Roijen, Van Straten, Al, Rutten, & Donker, 2006). This is a 27-item self-report questionnaire, assessing both direct and indirect costs of psychiatric illness. This will be administered as an interview.

### Sample size

Given previous research studies carried out at with this population, it is reasonable to assume that the following numbers will be applicable for this study (Shalev et al., 2011). Over 18 months, there will be 600 valid cases (that match the aforementioned inclusion and exclusion criteria). Of these, approximately 33% will have sufficient symptoms at 2 weeks posttrauma. This will give 200 valid cases, and assuming 50% attrition rate, this will leave 100 subjects, 50 in the treatment group and 50 in the waitlist control group. Assuming 30% spontaneous recovery rates in the waitlist group and 70% recovery rate in the Internet intervention, an alpha of 0.05 and power of 0.95, a total group size of 64 is needed.

### Data analysis

Data will be stored without identifying variables. It will be analyzed using SPSS 21.0 (SPSS Inc., Chicago, IL). Descriptive statistics will be used to describe the sample in terms of demographic profiles. The main outcome variable is PTSD diagnosis according to the CAPS, at 12 months posttreatment. Secondary outcomes include the severity of PTSD symptoms and depression symptoms.

The statistical analyses will be performed on the data of those participants who had participated in the pretreatment telephonic interview and in at least one follow-up wave. The principle method of data analysis will be a conditional latent class growth modeling with covariates (Bollen & Curran, 2006) using the Mplus version 7.2 software (Muthén & Muthén, 1998–2011). This method allows for comparing the trajectories of change in each of the five outcome variables between the two experimental groups while considering for possible preexisting differences in background characteristics. If the trial results are compatible with the research hypotheses, we expect the intercepts (the initial levels) of the growth curves in both groups to be identical and the slopes in the intervention group to be steeper than in the control group, indicating larger improvement in symptoms in the former group. We will use an estimation method such as full-information maximum likelihood to take advantage of all available data in presence of missing values. In case that the data will be non-normally multivariately distributed, as tested by Mardia's statistics, we will use estimation with robust standard errors (Little & Rubin, 2002) and corrected chi-square values. In the analyses, appropriate adjustments will be made for the permuted-block design of the trial sample.

## Discussion

This study is designed to contribute to the literature on PTSD interventions in several ways. First, it allows for Internet-based assessments. Although this method is frequently used in research, it is less common in intervention research. Should it prove helpful, with high uptake, it will reduce the costs of outreach and assessment in the immediate aftermath of a traumatic event. Since studies have repeatedly shown that early interventions for PTSD are helpful only in those who are symptomatic, accurate assessment is salient. Second, as described earlier, most Internet-based interventions rely on patients logging in independently. Some have reported low uptake. As far as these authors are aware, this is the first study to examine a patient–therapist combined Internet-based intervention, as an early intervention following trauma. The study will show whether this format leads to increased uptake. Third, although the use of VR in PTSD treatment has been established, it has not been used as an early treatment, and also not used in an online environment. This will allow for free use of the technology between sessions and the amount of time spent on these tasks will be examined.

Several potential limitations to this study may be encountered. First, although previous studies have demonstrated high compliance with initial telephone interviewing in this population, the way in which this will translate to Internet-based assessment is not known. Second, the uptake of such a novel treatment remains to be tested. Third, this study relies on clinical interviews conducted by telephone; although these are considered reliable and valid, they may still differ from face-to-face assessments. Fourth, the strict entry requirements to the study, including an ability to use the Internet, will limit the generalizability of the results. Finally, this mode of delivery for CBT has not been examined.

However, should this study prove to be effective and efficacious, then it may pave the way for Internet-based interventions that use new technology. Given the decreased costs of VR equipment, mostly in the non-therapeutic gaming environment, it seems probable that over the next several years, this mode of treatment will become more accessible, acceptable, and cost-effective.

## Supplementary Material

Early intervention for preventing posttraumatic stress disorder: an Internet-based virtual reality treatment

Early intervention for preventing posttraumatic stress disorder: an Internet-based virtual reality treatment

Early intervention for preventing posttraumatic stress disorder: an Internet-based virtual reality treatment

Early intervention for preventing posttraumatic stress disorder: an Internet-based virtual reality treatment

## References

[CIT0001] American Psychiatric Association (2013). DSM 5. American Journal of Psychiatry.

[CIT0002] Beck A, Steer R, Brown G (1996). Manual for the Beck Depression Inventory-II (BDI-II).

[CIT0003] Bisson J. I, Ehlers A, Matthews R, Pilling S, Richards D, Turner S (2007). Psychological treatments for chronic post-traumatic stress disorder: Systematic review and meta-analysis. British Journal of Psychiatry.

[CIT0004] Bollen K. A, Curran P. J, Bollen K. A, Curran P. J (2006). Nonlinear trajectories and the coding of time. Latent curve models: A structural equation perspective.

[CIT0005] Bryant R, Sackville T, Dang S, Moulds M, Guthrie R (1999). Treating acute stress disorder: An evaluation of cognitive behavior therapy and supportive counseling techniques. The American Journal of Psychiatry.

[CIT0006] Bryant R. A, Moulds M, Nixon R. V. D (2003). Cognitive behaviour therapy of acute stress disorder: A four-year follow-up. Behaviour Research and Therapy.

[CIT0007] Difede J, Cukor J, Jayasinghe N, Patt I, Jedel S, Spielman L (2007). Virtual reality exposure therapy for the treatment of posttraumatic stress disorder following September 11, 2001. Journal of Clinical Psychiatry.

[CIT0008] Difede J, Hoffman H. G (2002). Virtual reality exposure therapy for World Trade Center post-traumatic stress disorder: A case report. Cyberpsychology & Behavior.

[CIT0009] First M. B, Spitzer R. L, Gibbon M, Williams J. B. W (1996). Structured Clinical Interview for DSM-IV Axis I Disorders, Clinician Version (SCID-CV).

[CIT0010] Freedman S, Hoffman H, Garcia-Palacios A, Tamar Weiss P, Avitzour S, Josman N (2010). Prolonged exposure and virtual reality-enhanced imaginal exposure for PTSD following a terrorist bulldozer attack: A case study. Cyberpsychology, Behavior and Social Networking.

[CIT0011] Freedman S. A, Brandes D, Peri T, Shalev A (1999). Predictors of chronic post-traumatic stress disorder. A prospective study. The British Journal of Psychiatry: The Journal of Mental Science.

[CIT0012] Hoge C. W, Castro C. A, Messer S. C, McGurk D, Cotting D. I, Koffman R. L (2004). Combat duty in Iraq and Afghanistan, mental health problems, and barriers to care. The New England Journal of Medicine.

[CIT0013] Kazdin A. E, Blase S. L (2011). Rebooting psychotherapy research and practice to reduce the burden of mental illness. Perspectives on Psychological Science.

[CIT0014] Kearns M. C, Ressler K. J, Zatzick D, Rothbaum B. O (2012). Early interventions for PTSD: A review. Depression and Anxiety.

[CIT0015] Klein B, Mitchell J, Abbott J, Shandley K, Austin D, Gilson K (2010). A therapist-assisted cognitive behavior therapy internet intervention for posttraumatic stress disorder: Pre-, post- and 3-month follow-up results from an open trial. Journal of Anxiety Disorders.

[CIT0016] Knaevelsrud C, Maercker A (2010). Long-term effects of an internet-based treatment for posttraumatic stress. Cognitive Behaviour Therapy.

[CIT0017] Lange A, Rietdijk D, Hudcovicova M, Van de Ven J.-P, Schrieken B, Emmelkamp P. M. G (2003). Interapy: A controlled randomized trial of the standardized treatment of posttraumatic stress through the internet. Journal of Consulting and Clinical Psychology.

[CIT0018] Lisetti C, Pozzo E, Lucas M, Hernandez F, Silverman W, Kurtines B (2009). Second life, bio-sensors, and exposure therapy for anxiety disorders. Annual Review of CyberTherapy and Telemedicine.

[CIT0019] Little R. J. A, Rubin D. B (2002). Statistical analysis with missing data.

[CIT0020] Meyerbröker K, Emmelkamp P. M. G (2010). Virtual reality exposure therapy in anxiety disorders: A systematic review of process-and-outcome studies. Depression and Anxiety.

[CIT0021] Mouthaan J, Sijbrandij M, De Vries G.-J, Reitsma J. B, Van de Schoot R, Goslings J. C (2013). Internet-based early intervention to prevent posttraumatic stress disorder in injury patients: Randomized controlled trial. Journal of Medical Internet Research.

[CIT0022] Muthén L. K, Muthén B. O (1998–2011). Mplus user's guide.

[CIT0023] Rothbaum B. O, Hodges L. F, Ready D, Graap K, Alarcon R. D (2001). Virtual reality exposure therapy for Vietnam veterans with posttraumatic stress disorder. Journal of Clinical Psychiatry.

[CIT0024] Ruwaard J, Broeksteeg J, Schrieken B, Emmelkamp P, Lange A (2010). Web-based therapist-assisted cognitive behavioral treatment of panic symptoms: A randomized controlled trial with a three-year follow-up. Journal of Anxiety Disorders.

[CIT0025] Shalev A. Y, Ankri Y, Israeli-Shalev Y, Peleg T, Adessky R, Freedman S (2012). Prevention of posttraumatic stress disorder by early treatment: Results from the Jerusalem trauma outreach and prevention study. Archives of General Psychiatry.

[CIT0026] Shalev A. Y, Ankri Y. L. E, Peleg T, Israeli-Shalev Y, Freedman S (2011). Barriers to receiving early care for PTSD: Results from the Jerusalem trauma outreach and prevention study. Psychiatric Services (Washington, D.C.).

[CIT0027] Sijbrandij M, Olff M, Reitsma J. B, Carlier I. V. E, De Vries M. H, Gersons B. P. R (2007). Treatment of acute posttraumatic stress disorder with brief cognitive behavioral therapy: A randomized controlled trial. American Journal of Psychiatry.

[CIT0028] Van Roijen L. H, Van Straten A, Al M, Rutten F, Donker M (2006). Cost-utility of brief psychological treatment for depression and anxiety. The British Journal of Psychiatry: The Journal of Mental Science.

[CIT0029] Weathers F. W, Blake D. D, Schnurr P. P, Kaloupek D. G, Marx B. P, Keane T. M (2013). The Clinician-Administered PTSD Scale for DSM-5 (CAPS-5).

[CIT0030] Weathers F. W, Litz B. T, Keane T. M, Palmieri P. A, Marx B. P, Schnurr P. P (2013). The PTSD checklist for DSM-5 (PCL-5).

