# Supplementary material for: Early intervention for preventing posttraumatic stress disorder: an Internet-based virtual reality treatment
Source: Eur J Psychotraumatol. 2015 Apr 2;6:10.3402/ejpt.v6.25608. doi: 10.3402/ejpt.v6.25608 (PMC4385907; doi:10.3402/ejpt.v6.25608)
Supplement: Early intervention for preventing posttraumatic stress disorder: an Internet-based virtual reality treatment [file EJPT-6-25608-s001.pdf]

Frühintervention zur Vorbeugung einer posttraumatischen Belastungsstörung: eine Internet-basierte Behandlung in virtueller Realität

Sara A Freedman, Ehud Dayan, Yael Bleich Kimelman, Heidi Weissman, Renana Eitans

Hintergrund: Bei etwa 20% der Personen, die einem traumatischem Ereignis ausgesetzt waren, entwickelt sich eine posttraumatische Belastungsstörung (PTBS). Studien haben gezeigt, dass die kognitive Verhaltenstherapie für die Behandlung chronischer posttraumatischer Belastungsstörungen wirksam ist. Es hat sich auch gezeigt, dass es möglich ist, eine PTBS zu verhindern, wenn man frühzeitig nach dem traumatischen Erlebnis interveniert. Studien haben jedoch gezeigt, dass die Annahme einer solchen frühen Behandlung im Allgemeinen nur sehr gering ist und es daher notwendig ist, Interventionen durch andere Medien bereitzustellen. Die Verwendung von Technologie kann eventuell diese Barrieren in der Behandlung überwinden.
Ziel: Dieser Artikel beschreibt eine randomisierte, kontrollierte Studie die eine frühzeitige Intervention zur kognitiven Verhaltenstherapie bei PTBS untersucht. Die Behandlung involviert eine „Virtuelle Realität" als Methode für die Bereitstellung von Stimulusmaterialien als Elemente der Behandlung. Die Intervention basiert auf einer Onlineeinheit, bei der sich TherapeutIn mit KlientIn über eine gesicherten Online-Plattform austauscht. Diese Seite beeinhaltet auch Multimedia-Komponenten, wie z.B. Videos oder Audiodateien, auf die die KlientIn auch zwischen den Sitzungen zugreifen kann.
Methode: 200 PatientInnen, die eine Level 1 Notaufnahme nach einem Autounfall erreichten, wurden nach dem Zufallsprinzip zu Behandlungs- oder Kontrollgruppen zugeordnet. Einschlusskriterien sind: 18-65 Jahre alt, PTBS-Symptome zwei Wochen im Beziehung mit dem aktuellen Trauma, keine Suizidalität, keine Psychosen. Die PatientInnen wurden zu vier Zeitpunkten telefonisch befragt, den InterviewerInnen war nicht bekannt zu welcher Gruppe die GesprächspartnerInnen gehörten: vor und nach der Behandlung, und 6 und 12 Monate nach der Behandlung. Die primäre Erkenntnis ist die PTBS-Symptomatik im zeitlichen Verlauf. Weitere Ergebnisse gibt es zur Depression und Kostenwirksamkeit.
Schlussfolgerung: Die Ergebnisse geben mehr Einblick in Auswirkungen der Vorsorgemaßnahmen im Allgemeinen und internetbasierten Frühinterventionen bei PTBS während der akuten Phase nach einem Traum. Mögliche Stärken und Schwächen werden dabei diskutiert.

Keywords/Schlagwörter: Internet-basierte Therapie, kognitive V erhaltenstherapie, PTSD, Frühintervention

Name of translator: Iris Fischer, Brigitte Lueger-Schuster European

Citation: European Journal of Psychotraumatology 2015, 6: 25608 - http://dx.doi.org/10.3402/ejpt.v6.25608
