# Supplementary material for: Early intervention for preventing posttraumatic stress disorder: an Internet-based virtual reality treatment
Source: Eur J Psychotraumatol. 2015 Apr 2;6:10.3402/ejpt.v6.25608. doi: 10.3402/ejpt.v6.25608 (PMC4385907; doi:10.3402/ejpt.v6.25608)
Supplement: Early intervention for preventing posttraumatic stress disorder: an Internet-based virtual reality treatment [file EJPT-6-25608-s002.pdf]

Travma sonrası stres bozukluğunun önlenmesinde erken müdahale: İnternet tabanlı sanal gerçeklik tedavisi

Sara A Freedman, Ehud Dayan, Yael Bleich Kimelman, Heidi Weissman, Renana Eitan

Arkaplan: TSSB travmaya maruz kalan kişilerin %20'sinde gelişir ve araştırmalar BDT'nin kronik travma sonrası stres bozukluğu (TSSB) için etkili bir tedavi olduğunu göstermiştir. Ayrıca, travmatik bir olay sonrası erken uygulandığında TSSB'yi önlediği de gösterilmiştir. Ancak, araştırmalar erken tedavi alımının genellikle düşük olduğunu göstermiştir ve bu sebeple başka ortamlarda müdahale sağlanmasına dair ihtiyaçlar belirlenmiştir. Teknolojinin kullanımı ile tedavinin önündeki engellerin üstesinden gelinebilir.
Amaç: Araştırma, TSSB için erken BDT müdahalesini inceleyen bir randomize kontrol çalışmasını (RCT) anlatmaktadır. Tedavi, Sanal Gerçekliği tedavinin maruz bırakma tabanlı unsurlarını sunma yöntemi olarak dahil etmiştir. Müdahale internet tabanlıdır, terapist ve hasta güvenli bir online sitede buluşacaktır. Bu site ayni zamanda, seanslar arasında hasta tarafından ulaşılabilen, tedavinin multimedya ögelerini (vidyo, odiyo, sanal gerçeklik gibi) de içerecektir.
Yöntem: Motorlu araç kazasını takiben 1. Seviye Acil Servis bölümüne (ER) getirilen 200 hasta seçkisiz bir şekilde tedavi ya da kontrol gruplarına seçilmiştir. Dahil edilme kriterleri: 18-65 yaş arasında olma, şimdiki travma ile ilgili travmadan sonraki iki haftadaki TSSB belirtileri, intihar eğilimi olmaması, psikoz olmaması. Hastalar araştırmaya kör olan bir takım tarafından dört farklı zamanda telefon ile değerlendirileceklerdir: tedaviden önce ve sonra ve tedaviden 6 ve 12 ay sonra. Birincil sonuç takip süresindeki TSSB belirtileridir. İkincil sonuçlar depresyon ve maliyet etkinliğini içermektedir. Analizler tedavi amacına yönelik temellidir.
Tartışma: Sonuçlar, önleyici müdahalelerin genel olarak etkinliği ve internet tabanlı erken müdahalelerin yaralanma yaşamış popülasyonda travmayı takip eden akut dönemde, özellikle TSSB üzerindeki etkinliği üzerine daha çok iç görü sağlayacaktır. Olası güçlü yönlerini ve kısıtlamalarını tartışacağız.

Anahtar kelimeler: internet tabanlı terapi; bilişsel davranışçı terapi; TSSB; erken müdahale.

Name of translator: Emek Yuce Zeyrek-Rios

Citation: European Journal of Psychotraumatology 2015, 6: 25608 - http://dx.doi.org/10.3402/ejpt.v6.25608
