# Supplementary material for: Early intervention for preventing posttraumatic stress disorder: an Internet-based virtual reality treatment
Source: Eur J Psychotraumatol. 2015 Apr 2;6:10.3402/ejpt.v6.25608. doi: 10.3402/ejpt.v6.25608 (PMC4385907; doi:10.3402/ejpt.v6.25608)
Supplement: Early intervention for preventing posttraumatic stress disorder: an Internet-based virtual reality treatment [file EJPT-6-25608-s003.pdf]

Early Intervention for Preventing Posttraumatic Stress Disorder: an Internet Based Virtual Reality Treatment

Sara A Freedman, Ehud Dayan, Yael Bleich Kimelman, Heidi Weissman, Renana Eitan

Wczesna interwencja kryzysowa w zapobieganiu zaburzeia po stresie traumatycznym: terapia z wykorzystaniem rzeczywistości wirtualnej Wprowadzenie: Badania wykazały, że około 20% ludzi eksponowanych na traumę cierpi potem z powodu PTSD. Jednocześnie wielokrotnie wykazano skuteczność terapii poznawczo- behawiorlanej w leczeniu chronicznego PTSD. Jednak skuteczność tego typu terapii w leczeniu pierwszej fazy PTSD nie jest do końca wysoka, stąd też coraz częściej wykorzystuje się nowe technologie w zapobieganiu PTSD zaraz po traumie.
Cel: Celem tej pracy było opisanie skuteczności wczesnej interwencji kryzysowej opartej o podejście poznawczo-bheawioralne wspomagane użyciem rzeczywistości wirtualnej wśród zrandomizowanej proby kontrolnej (ang. randomized controlled trial) Method: Zbadano 200 osób po traumie wypadku motorowego w wieku 18-65 lat. Kontrolowano nasielnie objawów PTSD, obecność tendencji samobójczych oraz stanów psychotycznych. Dyskusja: Wyniki niniejszych badań mogą dostarczyć wglądu w efektywność wczesnych interwencji kryzysowej w zapobieganiu PTSD, w tym wykorzystanie nowych technologii wirtualnych.

Słowa kluczowe: Terapia oparta na rzeczywistości wirtualnej; terapia poznawczo-behawiorlana; PTSD; wczesna interwencja kryzysowa

Name of translator: Marcin Rzeszutek, University of Finance and Management in Warsaw, Poland

Citation: European Journal of Psychotraumatology 2015, 6: 25608 - http://dx.doi.org/10.3402/ejpt.v6.25608
