# Supplementary material for: Early intervention for preventing posttraumatic stress disorder: an Internet-based virtual reality treatment
Source: Eur J Psychotraumatol. 2015 Apr 2;6:10.3402/ejpt.v6.25608. doi: 10.3402/ejpt.v6.25608 (PMC4385907; doi:10.3402/ejpt.v6.25608)
Supplement: Early intervention for preventing posttraumatic stress disorder: an Internet-based virtual reality treatment [file EJPT-6-25608-s004.pdf]

Раннее вмешательство для предотвращения посттравматического стрессового расстройства: лечение в Интернете на основе виртуальной реальности

Sara A Freedman, Ehud Dayan, Yael Bleich Kimelman, Heidi Weissman, Renana Eitan

История вопроса: Посттравматическое стрессовое расстройство (ПТСР) развивается примерно у 20% людей, подвергшихся воздействию травматического события. Для лечения хронического ПТСР была подтверждена эффективность когнитивно-поведенческой психотерапии (КПТ). Кроме того, она показана для предотвращения ПТСР при вмешательстве на ранних сроках после травматического события. Однако исследования показали, что эффективность в начале лечения, как правило, низка, и поэтому была определена необходимость обеспечения вмешательства с помощью других методов. Использование такой технологии может преодолеть барьеры на пути лечения.
Цель: Эта статья описывает рандомизированное контролируемое испытание, которое исследует эффективность ранней КПТ для ПТСР. В качестве метода доставки базовых элементов лечения используется виртуальная реальность. Вмешательство осуществляется на основе Интернета, так что терапевт и пациент "встретятся" на безопасном онлайн-сайте. Этот сайт будет также включать в себя мультимедийные компоненты лечения (например, видео, аудио, виртуальная реальность), которые могут быть доступны пациенту в период между сеансами.
Метод: 200 пациентов, поступивших в отделение скорой помощи в результате дорожно-транспортного происшествия, будут случайным образом включаться в группы либо для лечения, либо для контроля. Критериями включения являются: возраст 18-65 лет; симптомы ПТСР, связанные с текущей травмой, через две недели после ДТП; отсутствие суицидального поведения; отсутствие психоза. Пациенты будут оцениваться дистанционно по телефону, в четырех случаях: до и после лечения, через 6 и 12 месяцев после лечения. Первичный результат – отслеживание симптомов ПТСР. Вторичные результаты – отслеживают депрессию и экономическую эффективность. Анализ будет сделан на основании предполагаемого лечения.
Обсуждение: Результаты обеспечат более глубокое представление о последствиях для пострадавших проведенных профилактических мероприятий во время острой фазы после травмы относительно ПТСР в целом, и в начале вмешательства на основе Интернета, в частности. Мы будем обсуждать возможные достоинства и недостатки.

Ключевые слова: терапия на основе Интернета, когнитивно-поведенческая терапия, ПТСР, раннее вмешательство

Name of translator: Marina Scherbak

Citation: European Journal of Psychotraumatology 2015, 6: 25608 - http://dx.doi.org/10.3402/ejpt.v6.25608
